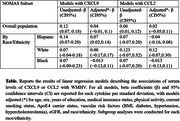# Race/Ethnicity Disparities in the Association of CXCL9 and CCL2 with the Burden of White Matter Disease: The Northern Manhattan Study

**DOI:** 10.1002/alz70856_102369

**Published:** 2025-12-24

**Authors:** Mohammad Nafeli Shahrestani, Hannah Gardener, Emir Veledar, Mitchell S.V. Elkind, Jose Gutierrez, Tatjana Rundek, Clinton B. Wright

**Affiliations:** ^1^ University of Miami Miller School of Medicine, Miami, FL, USA; ^2^ Evelyn F. McKnight Brain Institute, Miami, FL, USA; ^3^ Vagelos College of Physicians and Surgeons, Columbia University, New York, NY, USA; ^4^ Department of Epidemiology, Mailman School of Public Health, Columbia University, New York, NY, USA; ^5^ NINDS, Bethesda, MD, USA

## Abstract

**Background:**

Pro‐inflammatory cytokines and chemokines play a critical role in neurodegenerative diseases. However, the specific immunological pathways driving such pathology, and whether these are influenced by race or ethnicity, remain unclear. We wish to build on results reported in the prospective population‐based Northern Manhattan Study (NOMAS), showing a strong association between biomarkers CXCL9 (C‐X‐C Motif Chemokine Ligand 9), and CCL2 (C‐C Motif Chemokine Ligand 2) with incident mild cognitive impairment (MCI), and dementia. We examined associations of aforementioned biomarkers with the burden of cerebral white matter disease, measured by white matter hyperintensity volume (WMHV), an important risk factor for cognitive dysfunction, and whether the associations are influenced by race and ethnicity.

**Method:**

A subsample of clinically stroke‐free NOMAS participants underwent brain MRI, and WMHV was assessed quantitatively and divided by intracranial volume. CXCL9 and CCL2 were among 60 cytokines analysed from contemporaneous blood samples using a customized, 60‐plex, bead‐based immunoassay. The associations between the serum levels of these two proteins and WMHV were examined by two separate linear regression models using R, and adjusted for factors explained in the attached Table.

**Result:**

Participants (*n* = 1179) had a mean age of 69.8 ± 8.9 years; 60% were women, and 66.6% were Hispanic. Analysis of fully adjusted models revealed an association between CXCL9 and white matter hyperintensities (WMHV). This association was significantly modified by race/ethnicity, with the strongest effect observed in Hispanic participants (*p* value = 0.02). In contrast, CCL2 showed a weak association with WMHV, with no significant differences across racial/ethnic groups (*p* value = 0.34). Subgroup analyses, stratified by race/ethnicity, further supported these findings. In Hispanic participants, higher serum CXCL9 levels were significantly associated with increased WMHV (β per standard deviation (SD) = 0.075, *p* =  0.01). This association was weaker and not statistically significant in Black (β per SD =‐0.013, *p* = 0.81) or White (β per SD=0.003, *p* = 0.97) participants.

**Conclusion:**

Results of our study suggest interferon‐γ‐induced, angiostatic CXCL9 may be involved in the immune networks underlying leukoaraiosis. This association appears modified by ethnicity and may help explain the strong link between CXCL9 and cognitive impairment seen in our cohort.